# Prognostic value of Node-RADS scoring in stage IIICr cervical cancer: development and validation of novel nomograms

**DOI:** 10.1186/s13244-026-02222-7

**Published:** 2026-03-03

**Authors:** Li Jiang, Shanshan Ma, Qinghua Du, Jun Lv, Minghua Guo, Huisi Lin, Yanmei Que, Ting Gao, Shuxin Liang, Fang Wu, Yong Zhang

**Affiliations:** 1 Department of Radiation Oncology, The First Affiliated Hospital of Guangxi Medical University, Nanning, China; 2 Department of Toxicology, School of Public Health, Guangxi Medical University, Nanning, China; 3 Guangxi Colleges and Universities Key Laboratory of Prevention and Control of Highly Prevalent Diseases, Guangxi Medical University, Nanning, China; 4 State Key Laboratory of Targeting Oncology, Guangxi Medical University, Nanning, China; 5 Department of Radiation Oncology, The Second Affiliated Hospital of Guangxi Medical University, Nanning, China; 6 Department of Gynecology, The First Affiliated Hospital of Guangxi Medical University, Nanning, China; 7 Guangxi Medical University, Nanning, China

**Keywords:** FIGO stage IIICr cervical cancer, Node-RADS, Nomogram, Chemoradiotherapy, Prognosis

## Abstract

**Objectives:**

This study aimed to establish and validate nomograms integrating the Node-Reporting and Data System (Node-RADS) score and clinical variables to predict overall survival (OS) and progression-free survival (PFS) in patients with International Federation of Obstetrics and Gynecology (FIGO) 2018 stage IIICr cervical cancer receiving definitive chemoradiotherapy.

**Materials and methods:**

A retrospective two-center cohort study was conducted, enrolling eligible patients treated between March 2011 and December 2022. Nomograms were established based on least absolute shrinkage and selection operator (LASSO) regression, and Cox regression was used to identify prognostic features. The performance of the nomograms was assessed using receiver operating characteristic curves (ROC), calibration curves, and decision curve analysis (DCA).

**Results:**

A total of 307 eligible patients were analyzed. For OS, independent prognostic factors included para-aortic lymph node (PALN) metastasis, non-squamous histology, Node-RADS score, and lymph node (LN) boost irradiation ≥ 60 Gy EQD2 (Equivalent dose in 2Gy fractions); for PFS, these were T stage, non-squamous histology, and LN boost irradiation ≥ 60 Gy EQD2. Nomograms outperformed FIGO 2009/2018 staging in discrimination and clinical utility, with calibration curves showing good agreement between predicted and observed outcomes. Kaplan–Meier analysis linked higher Node-RADS scores, PALN metastasis, > 3 positive LNs, and LN boost irradiation < 60 Gy EQD2 to poorer OS and PFS.

**Conclusion:**

A nomogram incorporating the Node-RADS score, which is significantly associated with survival, can serve as a potential prognostic marker to assist clinicians in making informed decisions and developing individualized treatment strategies. Notably, the inherent treatment-selection bias in the retrospective design limits its direct therapeutic implications.

**Critical relevance statement:**

Node-RADS-based nomograms offer superior risk stratification and prognosis prediction for stage IIICr cervical cancer patients.

**Key Points:**

- The nomogram incorporating the Node-Reporting and Data System (Node-RADS) score can serve as a potential prognostic marker to assist clinicians.
- The nomogram incorporating the Node-RADS score, which integrated clinicopathological data and therapies, outperformed Federation of Obstetrics and Gynecology (FIGO) staging in discriminative ability and clinical utility.
- Higher Node-RADS scores correlated with worse survival outcomes of patients, and lymph node (LN) boost irradiation ≥ 60 Gy EQD2 might provide survival benefits.

**Graphical Abstract:**

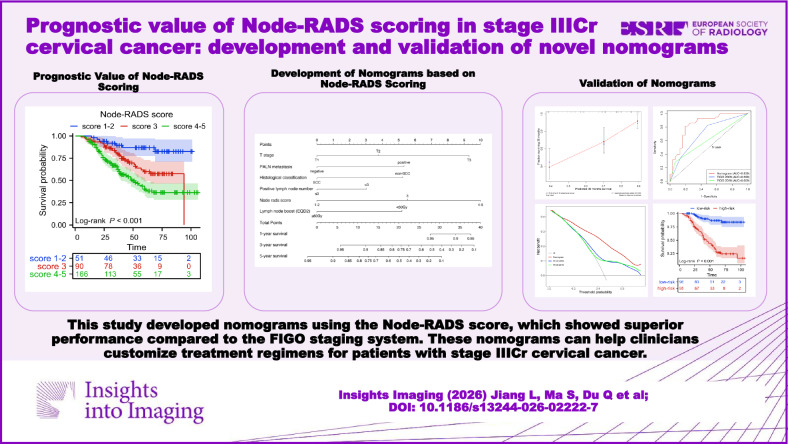

## Introduction

Cervical cancer is a common malignancy of the female reproductive system and the fourth most common cancer affecting women’s health [1]. The International Federation of Obstetrics and Gynecology (FIGO) 2018 staging system for cervical cancer, which uses radiology (r) and pathology (p) for staging, emphasizes the significance of lymph node (LN) metastasis and is frequently used for patient prognosis [2, 3]. The National Comprehensive Cancer Network (NCCN) Clinical Practice Guidelines in Oncology recommend concurrent chemoradiotherapy (CCRT) with brachytherapy for stage IIICr cervical cancer, but this approach does not fully account for LN traits and other prognostic factors [4]. Cross-sectional imaging exhibits suboptimal accuracy in predicting LN involvement, with performance varying based on criteria such as LN size and morphology. Studies have reported heterogeneous outcomes among patients with stage IIICr cervical cancer, and LN characteristics have been shown to be closely associated with prognosis [5, 6]. Therefore, there is an urgent need for a scoring system that comprehensively evaluates the characteristics of LN to predict the prognosis of patients with stage IIICr cervical cancer.

Currently, the Node-Reporting and Data System (Node-RADS) standardizes the radiological assessment of LN involvement in cancer using CT or MRI [7]. Node-RADS scores LNs according to size and configuration using a 5-point system, which captures nuanced lymph node characteristics beyond simple presence/absence defined by FIGO staging. While its utility has been validated in malignancies such as lung, bladder, and prostate cancer [8–10], the prognostic significance for cervical cancer remains inadequately elucidated. Nomograms visually predict cervical cancer prognosis [11, 12]. However, few nomograms can predict the prognosis of patients with stage IIICr cervical cancer undergoing definitive chemoradiotherapy. Thus, investigating the prognostic value of the Node-RADS score for these patients and creating precise and thorough models to predict prognosis is of critical clinical importance.

This study developed and validated nomograms combining the Node-RADS score and clinical variables to predict overall survival (OS) and progression-free survival (PFS) in patients with stage IIICr cervical cancer undergoing definitive chemoradiotherapy. Furthermore, we assessed the prognostic value of Node-RADS score and other factors in patients with stage IIICr cervical cancer.

## Materials and methods

### Patient selection

This retrospective two-center study enrolled patients with 2018 FIGO Stage IIICr cervical cancer who received definitive chemoradiotherapy at The First and Second Affiliated Hospitals of Guangxi Medical University between March 2011 and December 2022. The study protocol was approved by the Ethics Committee of The First and Second Affiliated Hospitals of Guangxi Medical University (2024-E531-01 and 2024-KY(0728)). Retrospective data were anonymized, and the requirement for informed consent was waived by the ethics committees. The inclusion criteria were as follows: (1) patients with cervical cancer diagnosed through pathology; (2) vaginal and rectal exploration performed; (3) patients restaged as IIICr on pelvic contrast-enhanced MRI (CE-MRI) and chest/abdominal contrast-enhanced CT (CE-CT) or whole-body PET/CT performed within 2 weeks before chemoradiotherapy; (4) patients receiving definitive chemoradiotherapy with or without adjuvant chemotherapy; and (5) complete clinicopathological and follow-up data. The exclusion criteria were as follows: (1) patients who had undergone other treatments before chemoradiotherapy, (2) complicated by other malignant diseases, and (3) incomplete clinicopathological and follow-up data. The patients were restaged by two oncologists using the 2018 FIGO system.

### MRI technique and image analysis

Patients underwent pelvic examination using a 3.0-T MRI scanner (GE Corporation) with a phased-array body coil. MRI protocols included standard sequences, and the main scanning parameters were used as previous study [13]. Node-RADS scoring in this study was performed by three senior radiologists with more than 10 years of experience in gynecological cancer. The Node-RADS score was determined independently by two radiologists (Y.P. and S.P.) using a three-level flowchart according to the original publication (Supplementary Fig. 1) [7]. When the two radiologists had different opinions, the third radiologist (M.L.J.) resolved disagreements. The image analysis was conducted in accordance with Node-RADS recommendations. Representative examples illustrating the various Node-RADS scores are displayed in Supplementary Fig. 2. Subcategory scores were based on the size and configuration criteria. The total score of the assessment categories ranged from 1 to 5 (1 = very low, 2 = low, 3 = equivocal, 4 = high, and 5 = very high), reflecting the likelihood of nodal involvement by the tumor.

### Treatment

All patients received pelvic external beam radiotherapy (EBRT) at a dose of 45–50.4 Gy plus brachytherapy with concurrent chemotherapy, of which 76 patients received adjuvant chemotherapy. Extended-field radiation was applied if metastasis was detected in the para-aortic LNs. EBRT planning was performed by using an intensity-modulated technique. Positive LNs received simultaneous additional dose escalations of 5–15 Gy. High-dose-rate brachytherapy was administered such that the cumulative dose of high-risk clinical tumor volume reached 80–85 Gy EQD2. The concurrent chemotherapy regimen was cisplatin-based (40 mg/m^2^ per week for five cycles or 100 mg/m^2^ per 3 weeks for two cycles). The adjuvant chemotherapy regimen comprised cisplatin plus docetaxel or 5-fluorouracil for two cycles.

### Follow-up

OS was defined as the time from the date of pathological diagnosis to the date of death or the last follow-up. PFS was defined as the interval from the date of pathological diagnosis to the date of disease progression or death or the most recent available follow-up. Follow-up assessments were scheduled every 4 months for the first 3 years after treatment completion, every 6 months for 3–5 years after radiotherapy, and annually after that. Follow-up visits included a comprehensive review of medical history, physical assessment, hematology and biochemistry analyses, pelvic CE-MRI, and chest/abdominal CE-CT or whole-body PET/CT examination when necessary.

### Construction of prognostic nomograms

Clinical data such as age, tumor stage, size, number of positive lymph nodes, histology, chemotherapy regimen, and LN boost dose were obtained from medical records and imaging. T stage, size, pelvic wall involvement, and LN metastasis status were determined via examination and imaging. Tumor sizes were defined as the greatest dimensions on physical examination and MRI/CT. Positive LNs, regarded as LN metastasis, were defined as LN measured ≥ 1.0 cm in the short axis on MRI/CT or after standardized uptake value on PET/CT of ≥ 2.5 [14–16]. Patients treated at The First Affiliated Hospital of Guangxi Medical University were assigned to the training cohort, while patients treated at The Second Affiliated Hospital of Guangxi Medical University were assigned to validation cohorts. The least absolute shrinkage and selection operator (LASSO)-based Cox regression model in R software version 4.0.1 was used to select the optimal prognostic factors. The “glmnet” R-package was used to establish the penalty coefficient and selection variables, and the five-fold cross-validation method was used to determine the penalty coefficient (λ) of the regression model. Applying this penalty reduced the most independent variable coefficients to zero, retaining only a few factors with nonzero weights. Hazard ratios (HRs) and 95% confidence intervals (CIs) for candidate prognostic factors were calculated. After identifying the optimal variables, a multivariate Cox regression analysis was performed. Features with a *p*-value < 0.20 in the multivariate Cox regression analysis or with clinical importance were included in the nomogram construction.

### Statistical analysis

In the training cohort, the optimal cutoff values for continuous and ordinal variables were calculated using X-tile software (version 3.6.1; Yale University). The χ^2^ test or the Fisher exact test was used to compare differences in categorical variables. The Kaplan–Meier method was used to assess OS and PFS using a log-rank test. The time-dependent receiver operating characteristic (tROC) curve, the corresponding area under the curve (tAUC), and the concordance index (C-index) were used to evaluate the discriminatory capacity of nomograms. Calibration curves were used to define predictive accuracy. Decision curve analysis (DCA) was used to assess the clinical utility of the nomogram. All statistical analyses were performed using SPSS (version 25.0) and R software (version 4.0.1) using “survival,” “rms,” and “timeROC” packages (http://www.r-project.org/). C-index was calculated using the concordance function from the survival package (version 3.7-0). Statistical significance was set at a two-tailed *p*-value < 0.05.

## Results

### Patient cohorts and clinicopathologic features

This retrospective two-center study analyzed 307 patients with stage IIICr cervical cancer who were treated at The First and Second Affiliated Hospitals of Guangxi Medical University. A total of 58 patients were excluded: 42 with incomplete medical records or follow-up data, 12 patients with prior treatment before chemoradiotherapy, and four due to complications from other malignant diseases. Of 307 patients, 191 from The First Affiliated Hospitals of Guangxi Medical University and 116 from The Second Affiliated Hospitals of Guangxi Medical University were assigned to the training and validation cohorts, respectively. The mean age of the entire cohort was 52.94 ± 12.72 years (range: 21–86). Additionally, 255 (83.1%) and 52 (16.9%) patients had FIGO stage IIIC1r and IIIC2r, respectively. Of the 307 patients, 76 (24.8%) received CCRT plus adjuvant chemotherapy, and 231 (75.2%) received CCRT alone. Most patients (248, 80.8%) had squamous histology. The positive LNs received a median irradiation dose of 52.08 (range, 45–65) Gy. The median follow-up periods were 42 (range, 5–106) and 39 (range, 8–89) months in the training and validation cohorts, respectively. The 5-year OS and PFS rates of the entire cohort were 54.3% and 49.4%, respectively. During follow-up, 143 patients (46.6%) progressed, and 122 (39.7%) died. The clinicopathological characteristics of patients with FIGO stage IIICr cervical cancer in the training and validation cohorts are summarized in Table 1.

**Table 1:** Clinicopathological characteristics of patients

Characteristics	Training cohort	Validation cohort	*p*-value
*n*	191	116	
Age, median (IQR)	51 (44, 62)	52.5 (45, 61)	0.627
T stage, *n* (%)			0.441
T1	45 (23.6%)	25 (21.6%)	
T2	110 (57.6%)	62 (53.4%)	
T3	36 (18.8%)	29 (25%)	
PALN metastasis, *n* (%)			0.912
Negative	159 (83.2%)	96 (82.8%)	
Positive	32 (16.8%)	20 (17.2%)	
Tumor differentiation, *n* (%)			0.028
Moderate	124 (64.9%)	60 (51.7%)	
Well	22 (11.5%)	25 (21.6%)	
Poor	45 (23.6%)	31 (26.7%)	
Histological classification, *n* (%)			0.699
SCC	153 (80.1%)	95 (81.9%)	
Non-SCC	38 (19.9%)	21 (18.1%)	
Primary tumor size, *n* (%)			0.598
≤ 4 cm	121 (63.4%)	70 (60.3%)	
> 4 cm	70 (36.6%)	46 (39.7%)	
Adjuvant chemotherapy, *n* (%)			0.640
No	142 (74.3%)	89 (76.7%)	
Yes	49 (25.7%)	27 (23.3%)	
Positive lymph node number, *n* (%)			0.565
≤ 3	136 (71.2%)	79 (68.1%)	
> 3	55 (28.8%)	37 (31.9%)	
Node-RADS score, *n* (%)			0.417
1	3 (1.6%)	4 (3.4%)	
2	27 (14.1%)	17 (14.7%)	
3	54 (28.3%)	36 (31%)	
4	84 (44%)	40 (34.5%)	
5	23 (12%)	19 (16.4%)	
Lymph node boost (EQD2), *n* (%)			0.172
≥ 60 Gy	69 (36.1%)	51 (44%)	
< 60 Gy	122 (63.9%)	65 (56%)	

*PALN* para-aortic lymph node, *IQR* interquartile range, *SCC* squamous cell carcinoma, *RADS* reporting and data system, *EQD2* equivalent dose in 2 Gy fractions

### Identification of prognostic features for OS and PFS

Univariate Cox regression analysis was performed to explore the predictive factors related to OS and PFS (Table 2). LASSO regression was used to reduce the effects of collinearity among parameters (Supplementary Fig. 3). Multivariate Cox regression analysis showed that para-aortic lymph node (PALN) metastasis (hazard ratio (HR) =1.8, *p* = 0.045), histological classification (HR = 1.79, *p* = 0.032), Node-RADS score (score 4–5 vs score 1–2; HR = 3.24, *p* = 0.028), and LN boost irradiation ≥ 60 Gy EQD2 (HR = 0.499, *p* = 0.022) were independent prognostic factors for OS (Table 3). Moreover, T stage (T2 vs T1: HR = 2.08, *p* = 0.026), histological classification (HR = 1.96, *p* = 0.006), and LN boost irradiation of ≥ 60 Gy EQD2 (HR = 0.48, *p* = 0.005) were independent prognostic factors for PFS (Table 3).

**Table 2:** Univariate Cox regression analysis of the training cohort for OS and PFS

Characteristics	Total (*N*)	OS		PFS	
		Hazard ratio (95% CI)	*p*-value	Hazard ratio (95% CI)	*p*-value
Age	191				
≤ 60	138	Reference		Reference	
> 60	53				
T stage	191				
T1	45	Reference		Reference	
T2	110	1.46 (0.76–2.81)	0.256	1.92 (1.05–3.51)	**0.035**
T3	36	4.56 (2.37–8.80)	**< 0.001**	4.53 (2.39–8.59)	**< 0.001**
PALN metastasis	191				
Negative	159	Reference		Reference	
Positive	32	3.29 (2.00–5.43)	**< 0.001**	2.50 (1.55–4.00)	**< 0.001**
Tumor differentiation	191				
Well	22	Reference		Reference	
Moderate	124	0.59 (0.30–1.14)	0.117	0.80 (0.42–1.52)	0.493
Poor	45	0.67 (0.32–1.41)	0.29	0.87 (0.42–1.78)	0.694
Histological classification	191				
SCC	153	Reference		Reference	
Non-SCC	38	2.23 (1.35–3.69)	**0.002**	2.14 (1.35–3.38)	**0.002**
Primary tumor size	191				
≤ 4 cm	121	Reference		Reference	
> 4 cm	70	2.91 (1.83–4.63)	**< 0.001**	2.52 (1.66–3.83)	**< 0.001**
Adjuvant chemotherapy	191				
No	142	Reference		Reference	
Yes	49	0.95 (0.56–1.60)	0.843	0.90 (0.56–1.46)	0.679
Node-RADS score	191				
1–2	30	Reference		Reference	
3	54	2.65 (0.88–7.92)	0.082	1.75 (0.80–3.8)	0.082
4–5	107	5.02 (1.81–13.90)	**0.002**	2.42 (1.2–4.89)	**0.029**
Positive lymph node number	191				
≤ 3	136	Reference		Reference	
> 3	55	2.18 (1.37–3.47)	**< 0.001**	1.83 (1.20–2.8)	**0.005**
Lymph node boost (EQD2)	191				
< 60 Gy	122	Reference		Reference	
≥ 60 Gy	69	0.56 (0.33–0.96)	**0.036**	0.59 (0.37–0.95)	**0.029**

*CI* confidence interval, *OS* overall survival, *PFS* progression-free survival, *PLAN* para-aortic lymph node, *SCC* squamous cell carcinoma, *RADS* reporting and data system, *EQD2* equivalent dose in 2 Gy fractions

The bold values indicate *p*-values 0.05, representing statistically significant differences

**Table 3:** Multivariate Cox regression analysis of the training cohort for OS and PFS

Characteristics	Total (*N*)	OS		PFS	
		Hazard ratio (95% CI)	*p*-value	Hazard ratio (95% CI)	*p*-value
Age	191				
≤ 60	138	Reference		Reference	
> 60	53				
T stage	191				
T1	45	Reference		Reference	
T2	110	1.42 (0.71–2.86)	0.322	2.08 (1.09–3.94)	**0.026**
T3	36	1.99 (0.71–5.60)	0.190	2.41 (0.98–5.92)	0.056
PALN metastasis	191				
Negative	159	Reference		Reference	
Positive	32	1.80 (1.01–3.20)	**0.045**	1.62 (0.96–2.73)	0.070
Histological classification	191				
SCC	153	Reference		Reference	
Non-SCC	38	1.79 (1.05–3.05)	**0.032**	1.96 (1.21–3.17)	**0.006**
Primary tumor size	191				
≤ 4 cm	121	Reference		Reference	
> 4 cm	70	1.54 (0.67–3.52)	0.309	1.59 (0.82–3.08)	0.172
Node-RADS score	191				
1–2	30	Reference		Reference	
3	54	1.94 (0.64–5.87)	0.239	1.26 (0.57–2.79)	0.566
4–5	107	3.24 (1.14–9.23)	**0.028**	1.65 (0.79–3.43)	0.184
Positive lymph node number	191				
≤ 3	136	Reference		Reference	
> 3	55	1.41 (0.85–2.33)	0.185	1.40 (0.86–2.22)	0.149
Lymph node boost (EQD2)	191				
< 60 Gy	122	Reference		Reference	
≥ 60 Gy	69	0.50 (0.28–0.91)	**0.022**	0.48 (0.29–0.81)	**0.005**

*CI* confidence interval, *OS* overall survival, *PFS* progression-free survival, *SCC* squamous cell carcinoma, *RADS* reporting and data system, *EQD2* equivalent dose in 2 Gy fractions, *PALN* para-aortic lymph node

The bold values indicate *p*-values 0.05, representing statistically significant differences

### Construction and validation of the nomogram for prognosis prediction

**Fig. 1:**
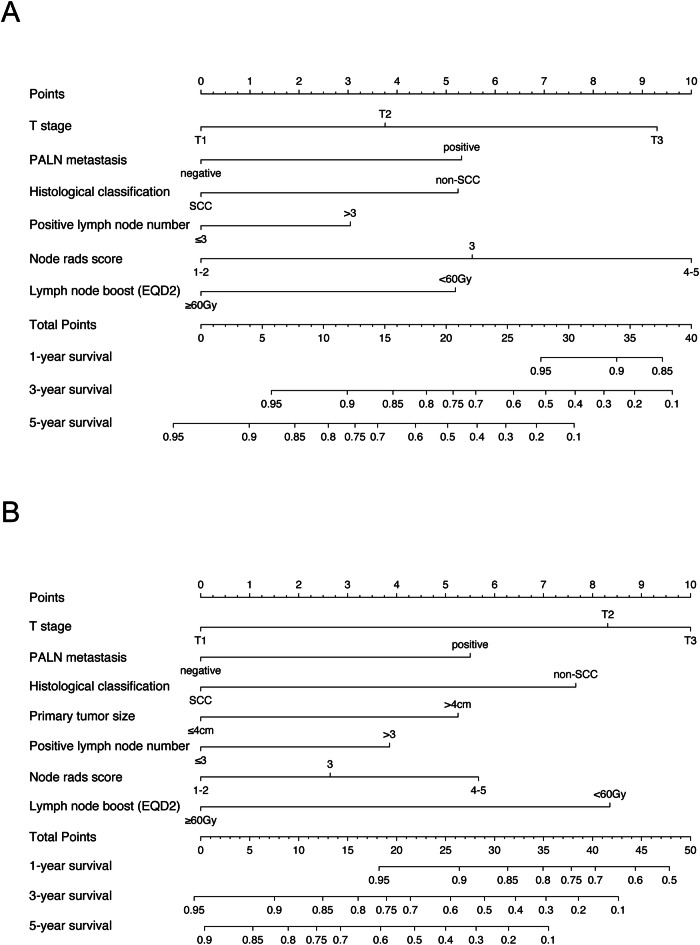
Nomogram for predicting OS (**A**) and PFS (**B**) in patients with FIGO stage IIICr cervical cancer. OS, overall survival; PFS, progression-free survival; FIGO, International Federation of Obstetrics and Gynecology; EQD2, Equivalent dose in 2 Gy fractions

**Fig. 2:**
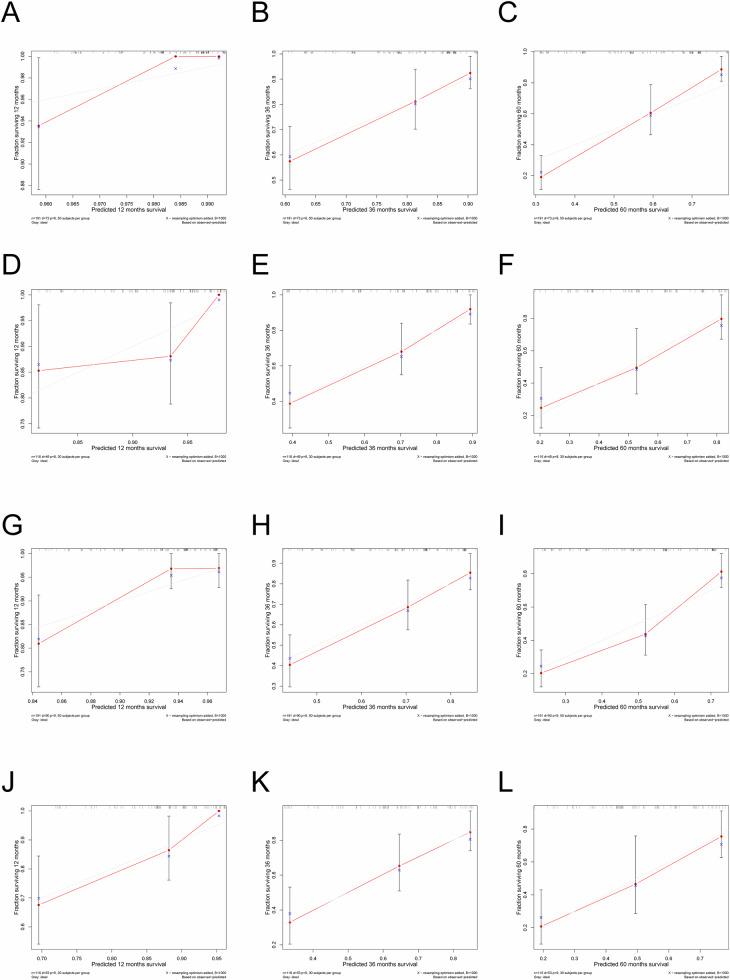
**A**–**C** Nomogram calibration curves for predicting the 1-, 3-, and 5-year OS in the training cohort. **D**–**F** Nomogram calibration curves for predicting the 1-, 3-, and 5-year OS in the validation cohort. **G**–**I** Nomogram calibration curves for predicting 1-, 3-, and 5- PFS in the training cohort. **J**–**L** Nomogram calibration curves for predicting the 1-, 3-, and 5-year PFS in the validation cohort. OS, overall survival; PFS, progression-free survival

**Fig. 3:**
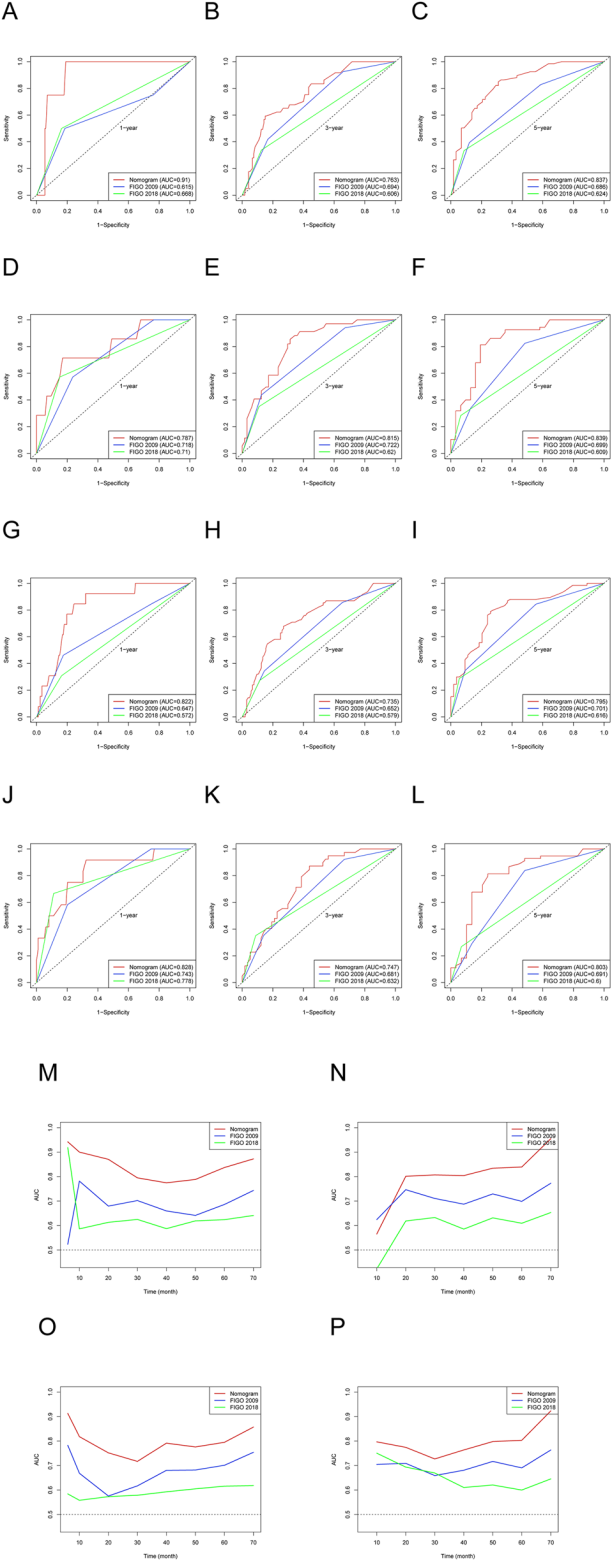
Time-dependent ROC analysis comparing the nomogram and FIGO staging system. **A**–**C** AUC for predicting the 1-, 3-, and 5-year OS in the training cohort. **D**–**F** AUC for predicting 1-, 3-, and 5-year OS in the validation cohort. **G**–**I** AUC for predicting the 1-, 3-, and 5-year PFS in the training cohort. **J**–**L** AUC for predicting 1-, 3-, and 5-year PFS in the validation cohort. Time-dependent AUC between 10 and 70 months for OS in the training (**M**) and validation (**N**) cohorts. Time-dependent AUC between 10 and 70 months for PFS in the training (**O**) and validation (**P**) cohorts. OS, overall survival; PFS, progression-free survival; ROC, receiver operating characteristic; AUC, area under the curve; FIGO, International Federation of Obstetrics and Gynecology

**Fig. 4:**
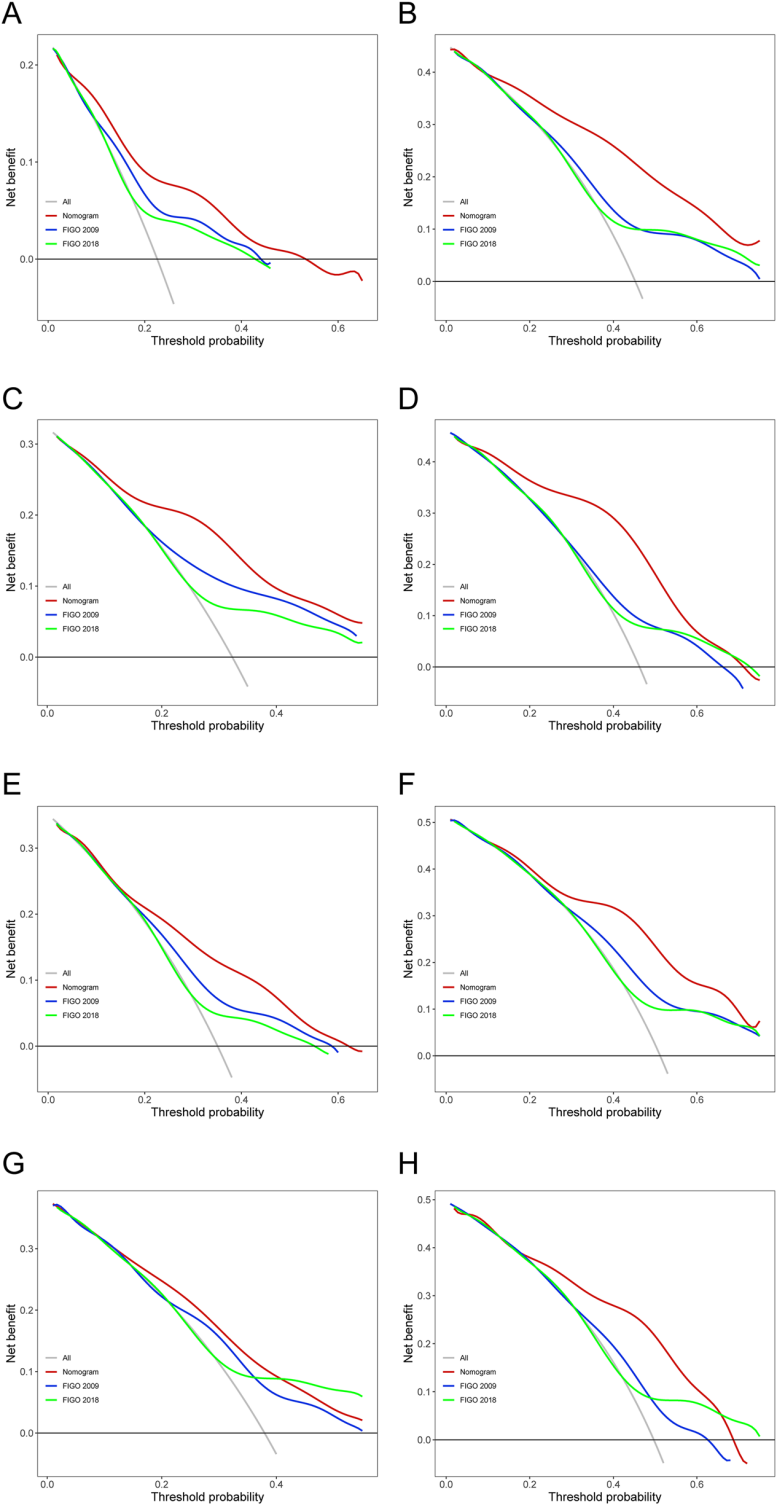
Decision curve analysis to compare the clinical utility of the nomograms and FIGO staging system for 3- and 5-year OS in the training (**A**, **B**) and validation (**C**, **D**) cohorts and 3- and 5-year PFS in the training (**E**, **F**) and validation (**G**, **H**) cohorts. OS, overall survival; PFS, progression-free survival; FIGO, International Federation of Obstetrics and Gynecology

Based on the above results of the prognostic factor selection, we constructed nomograms for predicting OS and PFS (Fig. 1). Calibration curves of the nomograms demonstrated a strong fit with the 45-degree line, indicating alignment between the predicted and observed outcomes for OS and PFS (Fig. 2). The nomogram for OS achieved the C-index of 0.775 and 0.750 in the training and validation cohorts, respectively, outperforming both FIGO 2009 and FIGO 2018 staging. Similarly, the PFS nomogram yielded C-indices of 0.717 (training cohort) and 0.702 (validation cohort), which were consistently higher than those of the FIGO staging systems. The tROC analysis of the nomograms showed AUC values for the 1-, 3-, and 5-year OS and PFS ranging from 0.735 to 0.910, which was consistently superior to those of the FIGO staging systems (Fig. 3). DCA further validated the clinical utility of the nomograms, showing that they provided greater net clinical benefits across a broad range of threshold probabilities for both OS and PFS prediction relative to the FIGO staging systems (Fig. 4).

### Risk stratification according to the nomograms

**Fig. 5:**
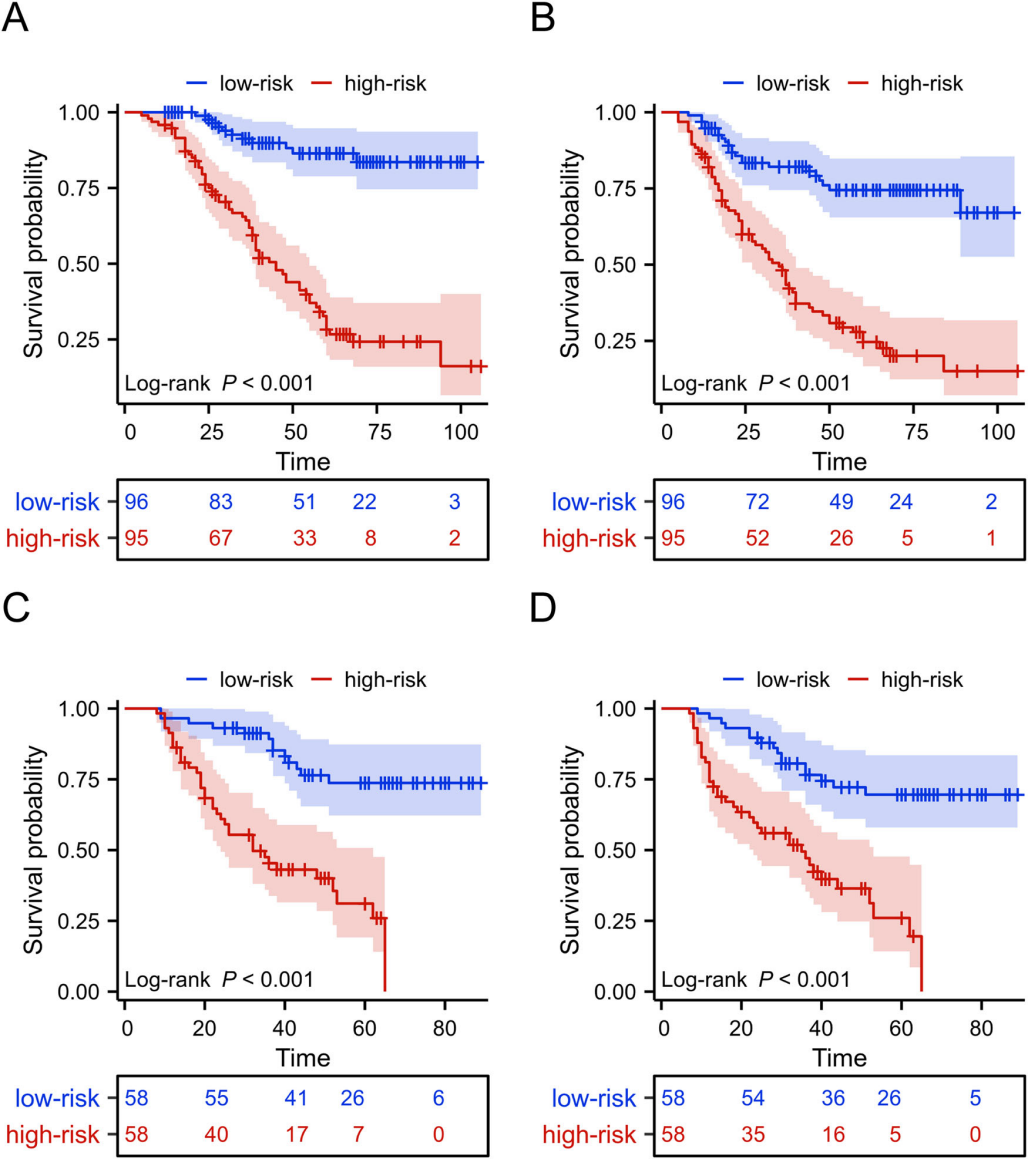
Kaplan–Meier curves of OS (**A**, **B**) and PFS (**C**, **D**) for high- and low-risk groups divided by nomogram-generated scores in the training and validation cohorts. OS, overall survival; PFS, progression-free survival

A risk stratification system was established by summing the scores derived from the nomograms, which categorized patients into high- and low-risk groups in both the training and validation cohorts. In the training cohort, this stratification significantly differentiated the Kaplan–Meier curves for 5-year OS (high-risk vs low-risk: 28.2% vs 86.4%, *p* < 0.001) and 5-year PFS (high-risk vs low-risk: 24.6% vs 74.5%, *p* < 0.001). The validation cohort showed similar trends (OS, high-risk vs low-risk: 25.9% vs 73.7%, *p* < 0.001; PFS, high-risk vs low-risk: 19.5% vs 69.5%, *p* < 0.001) (Fig. 5).

### Survival analysis based on Node-RADS score and boost irradiation

**Fig. 6:**
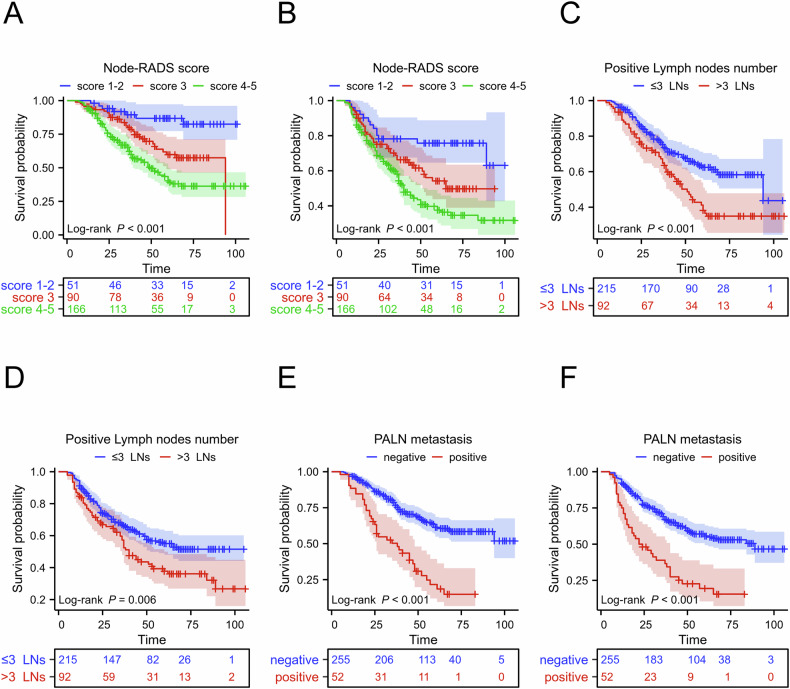
Kaplan–Meier analyses of the Node-RADS score (**A**, **B**), positive lymph node number (**C**, **D**), and PALN metastasis (**E**, **F**) in the entire cohort for OS and PFS. OS, overall survival; PFS, progression-free survival; Node-RADS, Node-Reporting and Data System; PALN, para-aortic lymph node

To assess the effect of the Node-RADS score on survival in the entire cohort, patients were divided into three groups (Node-RADS score 1–2 vs score 3 vs score 4–5, Fig. 6). Significant differences in 5-year OS were observed among these groups (86.8%, 59.8%, and 40.3%, respectively; *p* < 0.001). For PFS, a worse outcome was noted in higher-score groups, with a significant difference between the scores 4–5 and 1–2 (37.6% vs 75.7%, *p* < 0.001). Regarding the LN number, a cutoff value of 3 was used in the X-tile software. Patients with > 3 positive LNs had inferior 5-year OS (62.4% vs 38.1%, *p* < 0.001) and PFS (55.0% vs 37.7%, *p* = 0.006). PALN metastasis was also associated with poorer survival (5-year OS: 21.6% vs 61.4%, *p* < 0.001; 5-year PFS: 19.4% vs 55.7%, *p* < 0.001). Subgroup analyses revealed that higher Node-RADS scores correlated with worse OS and PFS only in patients with stage IIIC1r and patients with ≤ 3 positive LNs (Supplementary Fig. 4). Furthermore, the tROC analysis indicated that a combination of PALN metastasis and Node-RADS score increased the AUC values for OS and PFS with an average of 0.7 (Supplementary Fig. 5). As for the total dose of LN boost, a cutoff value of 60 Gy EQD2 was used in the X-tile software. Of the 307 patients, 120 received LN-boost irradiation of positive LNs (EQD2 ≥ 60 Gy). Patients with a LN boost dose ≥ 60 Gy had higher 5-year OS (66.6% vs 47.2%) and 5-year PFS (59.0% vs 43.6%) rates than those with an LN boost dose < 60 Gy (*p* < 0.01; Supplementary Fig. 6). For stage IIIC1 patients, an LN boost dose ≥ 60 Gy significantly increased the 5-year OS (71.3% vs 55.3%, *p* = 0.041) and PFS (65.4% vs 49.6%, *p* = 0.007), but not in stage IIIC2 patients. For patients with stage IIIC2 disease, an LN boost dose ≥ 55 Gy improved 5-year OS (33.49% vs 13.85%, *p* = 0.046) but not PFS (Supplementary Fig. 6). Moreover, an LN boost dose ≥ 60 Gy improved OS (55.4% vs 28.3%, *p* = 0.02) and PFS (51.8% vs 29.2%, *p* = 0.019) only in patients with > 3 positive LNs. Subgroup analysis by Node-RADS score showed that an LN boost dose ≥ 60 Gy only significantly improved survival in patients with Node-RADS scores of 5 (5-year OS: 46.8% vs 9.5%, *p* = 0.016; PFS: 46.7% vs 9.9%, *p* = 0.043, Supplementary Fig. 7). Additionally, Kaplan–Meier survival analysis of other predictive features is presented in Supplementary Fig. 8.

## Discussion

This study developed and validated novel nomograms incorporating the Node-RADS score to predict OS and PFS in patients with FIGO 2018 stage IIICr cervical cancer undergoing definitive chemoradiotherapy. Our key findings demonstrate that the Node-RADS score is an independent prognostic factor for OS, along with PALN metastasis, non-squamous histology, and LN boost irradiation ≥ 60 Gy EQD2. For PFS, significant predictors included T stage, non-squamous histology, and LN boost ≥ 60 Gy EQD2. The resulting nomograms demonstrated superior predictive accuracy, discriminative ability, and clinical utility compared with both FIGO 2009 and 2018 staging systems. By integrating imaging-based nodal assessment with key clinicopathological variables, our models provide a more personalized approach to risk stratification and treatment planning, thereby addressing the heterogeneity inherent in stage IIICr disease.

In survival analyses, higher Node-RADS scores were associated with worse OS in patients with stage IIICr cervical cancer, as shown by Kaplan–Meier curves. A similar trend was observed for PFS, although a significant difference was observed between scores of 4–5 and 1–2. Two factors may explain this finding. First, the limited statistical power due to the small sample size likely reduced the ability to detect differences in subgroup comparisons. Second, the Node-RADS scores of 3 category itself may encompass a heterogeneous population. By definition, a Node-RADS score of 3 indicates potential non-metastatic reactive or inflammatory changes, reflecting the limitations of morphological evaluation. Supporting this, Wu et al reported that Node-RADS accurately diagnoses LN metastasis with scores of 4–5, but scores as low as 1–2 still resulted in > 25% of patients being diagnosed with LN metastasis [17]. This suggests that some patients with a score of 3 may have tumor burdens comparable to those with scores of 1–2. For ambiguous cases, particularly those with Node-RADS scores of 1–3, PET/CT may serve as a valuable adjunct by providing functional metabolic information that complements morphological MRI and helps clarify borderline findings.

Notably, Node-RADS significantly impacts OS/PFS only in stage IIIC1r or patients with ≤ 3 positive LNs. This may be because widespread nodal disease (IIIC2r or > 3 LNs) overshadows the prognostic value of individual node morphology evaluated by Node-RADS due to its large metastatic volume or systemic nature. Conversely, in patients with limited nodal disease, a high Node-RADS score may better reflect underlying tumor aggressiveness and thus serve as a stronger predictor of outcome. This aligns with prior evidence: a preliminary study showed that Node-RADS improves diagnostic accuracy for LN metastasis in cervical cancer [18]. As the Node-RADS score increased, so did the LN metastasis rate. This finding is biologically plausible, as features scored higher in Node-RADS (e.g., larger size, irregular borders, central necrosis) are often associated with more aggressive tumor biology and higher metastatic burden. Previous studies predominantly used measurement of LNs size on CT/MRI as a prognostic factor. For example, Avci et al reported that LNs > 17 mm on MRI were likely metastatic [19], and Pinto et al found that pelvic LNs > 2.5 cm predicted worse OS in 62 patients with stage IIIC1r disease [15]. However, size alone is an imperfect criterion: benign LNs can exceed 1 cm due to inflammation or immune hyperreactivity [20, 21], and metastases may occur in nodes smaller than 1 cm [22, 23]. Indeed, Lsaji et al observed no significant difference in PFS between patients with radiographic LNs ≥ 1 cm versus < 1 cm [24]. MRI/CT can clearly show the size, shape, border, necrosis and texture of lymph nodes, but there is a lack of effective quantitative tools to evaluate lymph nodes comprehensively. Our research shows that Node-RADS can effectively incorporate these features into the scoring system, representing a more advanced approach. Moreover, its quantitative nature enhances communication between radiologists and oncologists. However, Node-RADS only reflects the most suspicious LN features by the highest score, failing to fully assess all LN metastases (e.g., location and quantity). In this study, PALN metastasis and > 3 positive LNs were linked to poorer OS and PFS, similar to previous studies [25–28]. Notably, combining Node-RADS with PALN status significantly improved risk stratification, underscoring the complementary roles of qualitative nodal features and anatomical extent of disease. This synergy suggests that future iterations of nodal scoring systems might benefit from incorporating locational information like PALN status directly.

According to the NCCN guideline, a LN boost dose of 54–63 Gy is recommended for grossly involved LNs in patients with stage IIICr cervical cancer, although its clinical benefit remains uncertain. A systematic review and meta-analysis found that the 5-year OS rate was significantly higher with LN boost irradiation than without irradiation (71% vs 56%) [29]. Pelvic IMRT with a median nodal boost of 55 Gy in 25 fractions had low regional recurrence and toxicity [30, 31]. However, some studies argue that LN boosting may be unnecessary [32], as doses of 54–60 Gy improved short-term tumor control but did not translate into prolonged OS or disease-free survival [33, 34]. Our findings showed that LN boost dose ≥ 60 Gy EQD2 was associated with significantly improved OS and PFS in the overall cohort, adding to the ongoing debate regarding optimal nodal irradiation. Subgroup analyses revealed this benefit was particularly pronounced in specific high-risk subgroups: stage IIIC1r, > 3 positive LNs, and critically, Node-RADS score of 5. Node-RADS 5 nodes likely harbor radioresistant or bulky disease requiring higher doses for effective sterilization, consistent with radiobiological principles. Previous studies indicated that a standard EBRT dose of 50–60 Gy may be inadequate for bulk LN sterilization [35, 36]. Conversely, the absence of survival benefit in patients with lower Node-RADS scores (1–4) highlights the potential for treatment de-escalation in those with less suspicious nodes, thereby reducing toxicity without compromising oncologic outcomes. It is important to note that LN boost dose was not a predefined primary endpoint of this study, and the observed association may be confounded by treatment-selection bias (e.g., clinicians may have selected higher doses for more bulky or morphologically aggressive nodes), which cannot be fully adjusted for in a retrospective setting. Thus, this finding should be interpreted as hypothesis-generating rather than definitive evidence for dose escalation.

Our study had several limitations. First, this was a small two-center retrospective study, and the findings should be further verified in a prospective multicenter study with a larger sample size. Second, although functional imaging (e.g., DWI or PET/CT) is commonly used alongside CT/MRI, Node-RADS currently excludes such modalities by design to maintain simplicity. Future studies could incorporate functional imaging, such as PET-CT parameters with Node-RADS scores, to potentially enhance the prognostic accuracy. Third, due to data and technical constraints, we could not incorporate advanced imaging analysis techniques, such as radiomics or AI-based approaches, into the nomogram. Nevertheless, our work provided a foundation for future studies to build more sophisticated, radiomics-enhanced tools. Finally, we did not assess interobserver agreement in Node-RADS scoring, limiting our ability to evaluate the reliability and generalizability of these findings.

## Conclusion

This study developed nomograms using the Node-RADS score, which showed superior performance compared to the FIGO staging system. These nomograms can help clinicians customize treatment regimens for patients with stage IIICr cervical cancer. LN characteristics correlated with OS and PFS; however, several limitations need to be addressed. Notably, the absence of quantitative interobserver agreement analysis for Node-RADS scoring is a major methodological weakness, as it hinders confirmation of consistent interpretation across radiologists. Additionally, the LN boost dose finding was exploratory rather than a predefined primary endpoint, and inherent treatment-selection bias in the retrospective design precludes definitive conclusions regarding dose escalation. Further prospective multicenter studies should include kappa statistics to validate the reliability of Node-RADS scoring, alongside incorporating functional imaging and radiomic features to enhance the nomogram’s robustness, and specifically verify whether LN boost irradiation ≥ 60 Gy EQD2 confers therapeutic benefit.

## ELECTRONIC SUPPLEMENTARY MATERIAL

Supplementary information

## Data Availability

All data generated or analyzed during this study are included in this article/supplementary material. Further inquiries should be directed to the corresponding authors.

## Abbreviations

AUC: Area under the curve
CCRT: Concurrent chemoradiotherapy
C-index: Concordance index
DCA: Decision curve analysis
EBRT: External beam radiotherapy
EQD2: Equivalent dose in 2 Gy fractions
FIGO: International Federation of Gynecology and Obstetrics
HR: Hazard ratios
LASSO: Least absolute shrinkage and selection operator
LN: Lymph node
Node-RADS: Node-Reporting and Data System
PALN: Para-aortic lymph node
PET: Position emission tomography
PFS: Progression-free survival
tROC: Time-dependent receiver operating characteristic
