# Supplementary material for: Prognostic value of Node-RADS scoring in stage IIICr cervical cancer: development and validation of novel nomograms
Source: Insights Imaging. 2026 Mar 3;17:64. doi: 10.1186/s13244-026-02222-7 (PMC12957706; doi:10.1186/s13244-026-02222-7)
Supplement: Supplementary file 1 — Supplementary information [file 13244_2026_2222_MOESM1_ESM.pdf]

Prognostic Value of Node-RADS Scoring in Stage IIICr Cervical Cancer: Development and Validation of Novel Nomograms

ELECTRONIC SUPPLEMENTARY MATERIAL

Supplementary Figure 1. Explanation of the Node-RADS scoring system with adaptations from the original source [7].

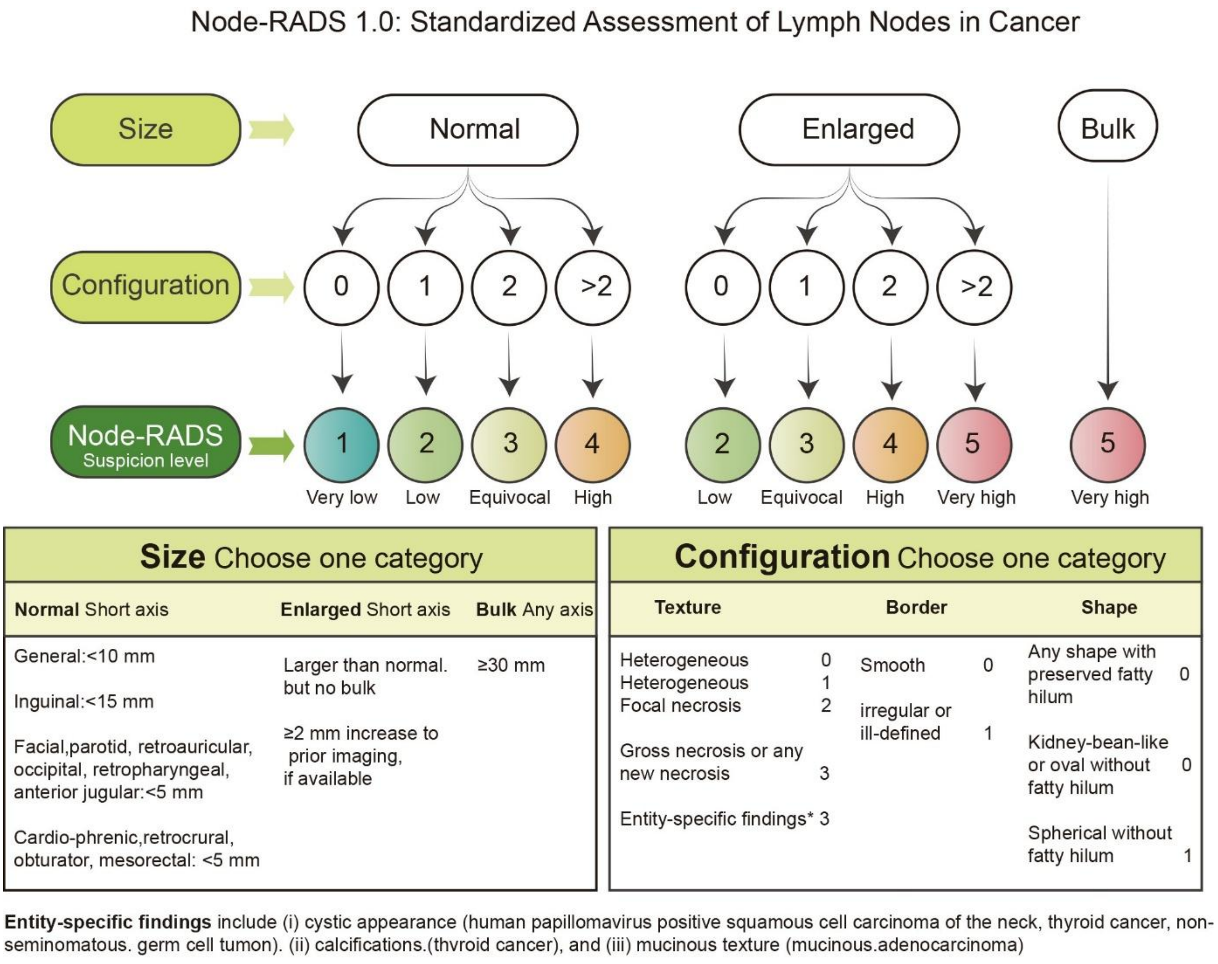

Node-RADS: Node-Reporting and Data System

Supplementary Figure 2. Representative examples illustrating Node-RADS scores.

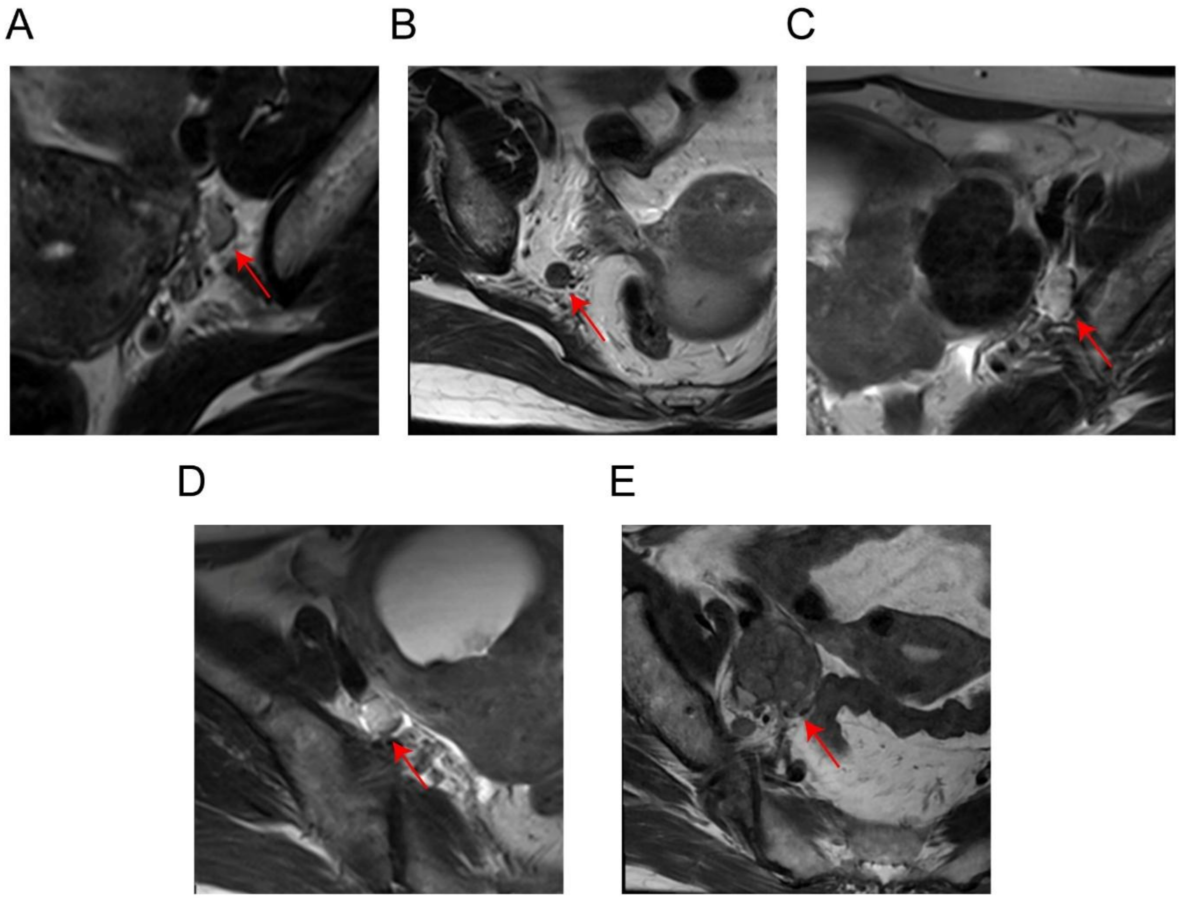

(**a**) Node-RADS score: 1; Features include a short axis measuring 9 mm (< 10mm), homogeneous texture (0 point), smooth borders (0 points), kidney-bean-like shape with fatty hilum (0 point). (**b**) Node-RADS score: 2; Features include a short axis measuring 7 mm (< 10mm), homogeneous texture (0 point), smooth borders (0 points), spherical shape without fatty hilum (1 point). (**c**) Node-RADS score: 3; Features include a short axis measuring 8 mm (< 10mm), heterogeneou texture (1 point), irregular borders (1 points), kidney-bean-like shape with fatty hilum (0 point). (**d**) Node-RADS score: 4; Features include a short axis measuring 12 mm (> 10mm), heterogeneous texture (1 point), smooth borders (0 points), spherical shape without fatty hilum (1 point). (**e**) Node-RADS score: 5; Features include a short axis measuring 36 mm (> 10mm), heterogeneous texture (1 point), irregular borders (1 points), spherical shape without fatty hilum (1 point).

Supplementary Figure 3. Variable selection using LASSO-Cox regression analysis. Selection of the most appropriate penalty parameter (λ) in the LASSO-Cox regression analysis via the minimum criteria for OS (**a** and **b**) and PFS (**c** and **d**).

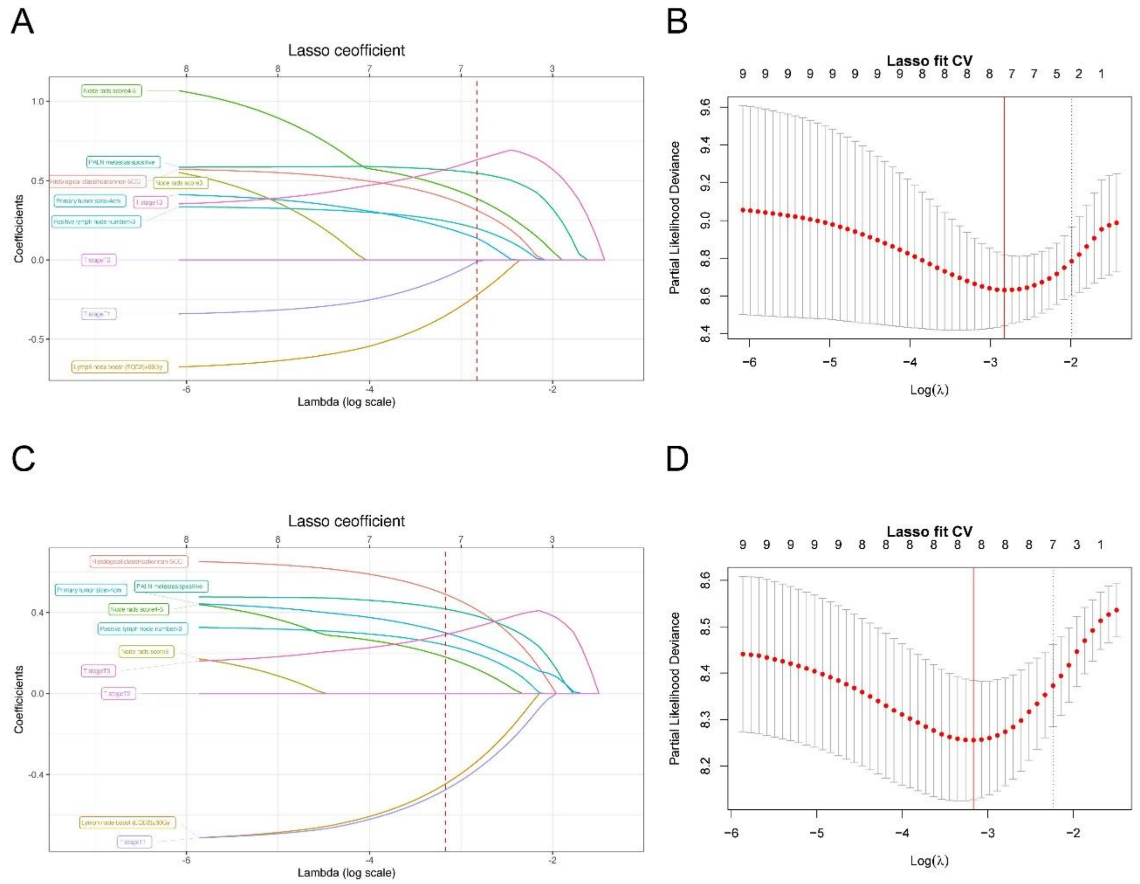

LASSO, least absolute shrinkage, and selection operator; OS, overall survival; PFS, progression-free survival

Supplementary Figure 4. Kaplan-Meier analyses of the Node-RADS scores in patients with FIGO stage IIIC1r (**a-b**), FIGO stage IIIC2r (**c-d**), ≤3 positive LNs (**e-f**), and >3 positive LNs (**g-h**) for OS and PFS.

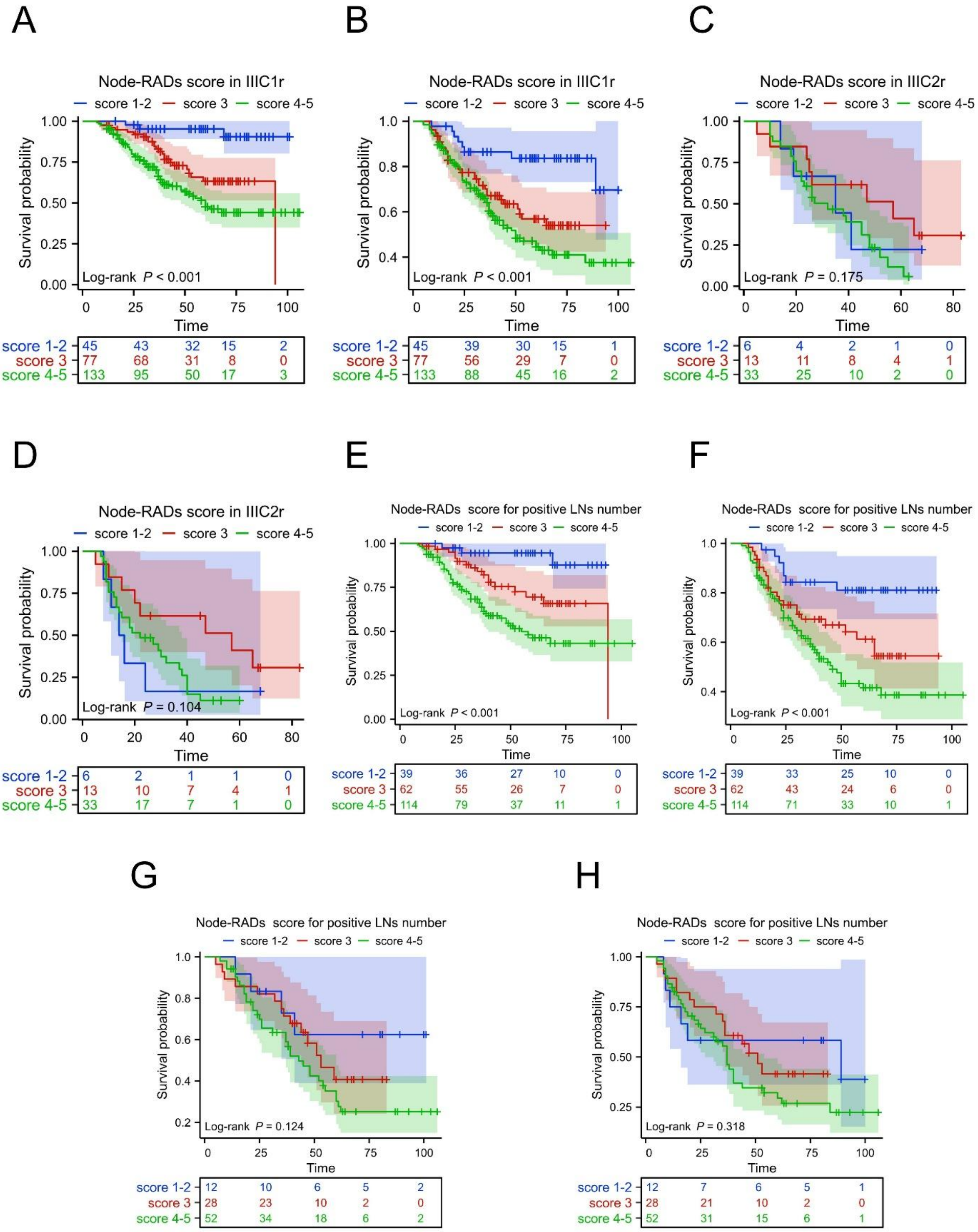

OS, overall survival; PFS, progression-free survival; FIGO, International Federation of Obstetrics and Gynecology; LN, lymph node; Node-RADS: Node-Reporting and Data System

Supplementary Figure 5. Time-dependent ROC analysis demonstrated that combining PALN metastasis and the Node-RADS score increased the AUC values for predicting 1-year (**a-b**), 3-year (**c-d**), and 5-year (**e-f**) OS and PFS.

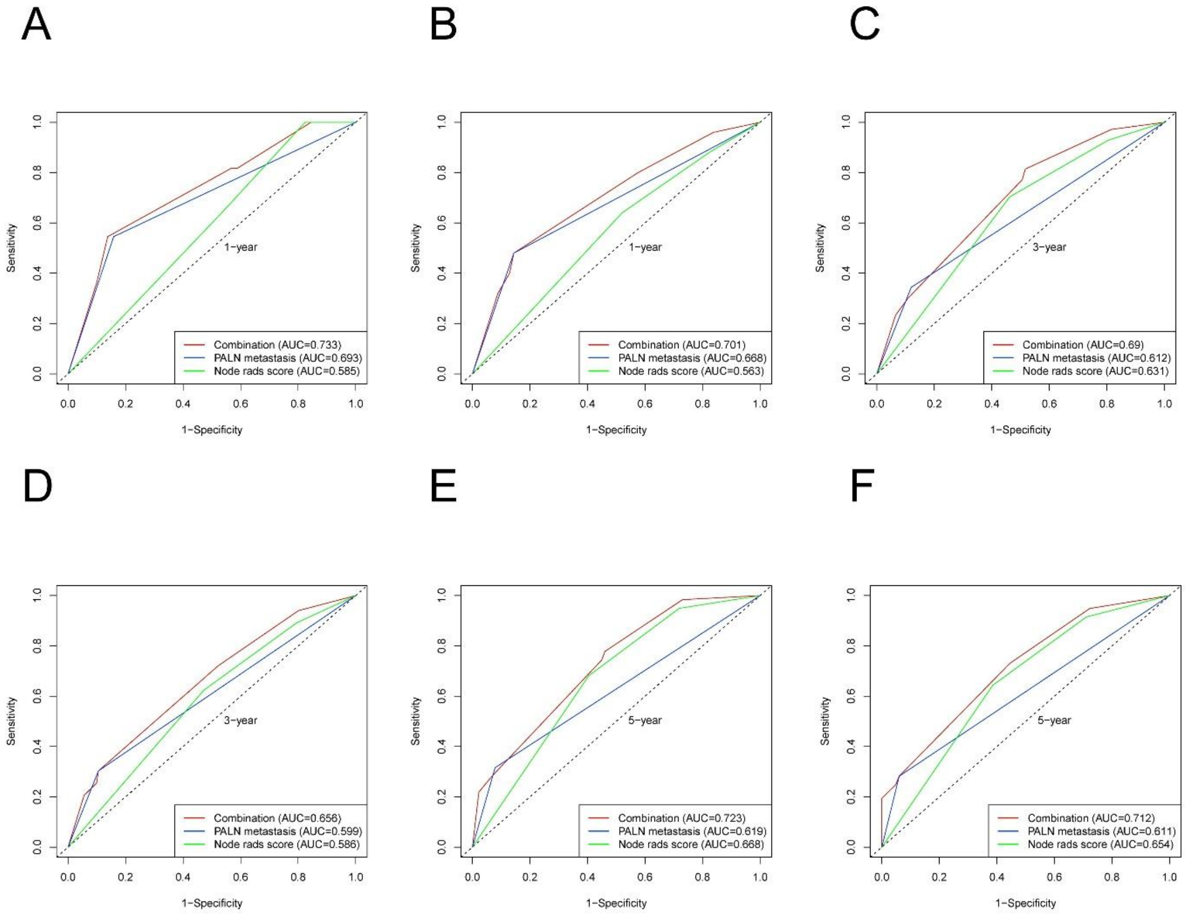

OS, overall survival; PFS, progression-free survival; ROC, receiver operating characteristic; AUC, area under the curve; Node-RADS: Node-Reporting and Data System; PALN: para-aortic lymph node

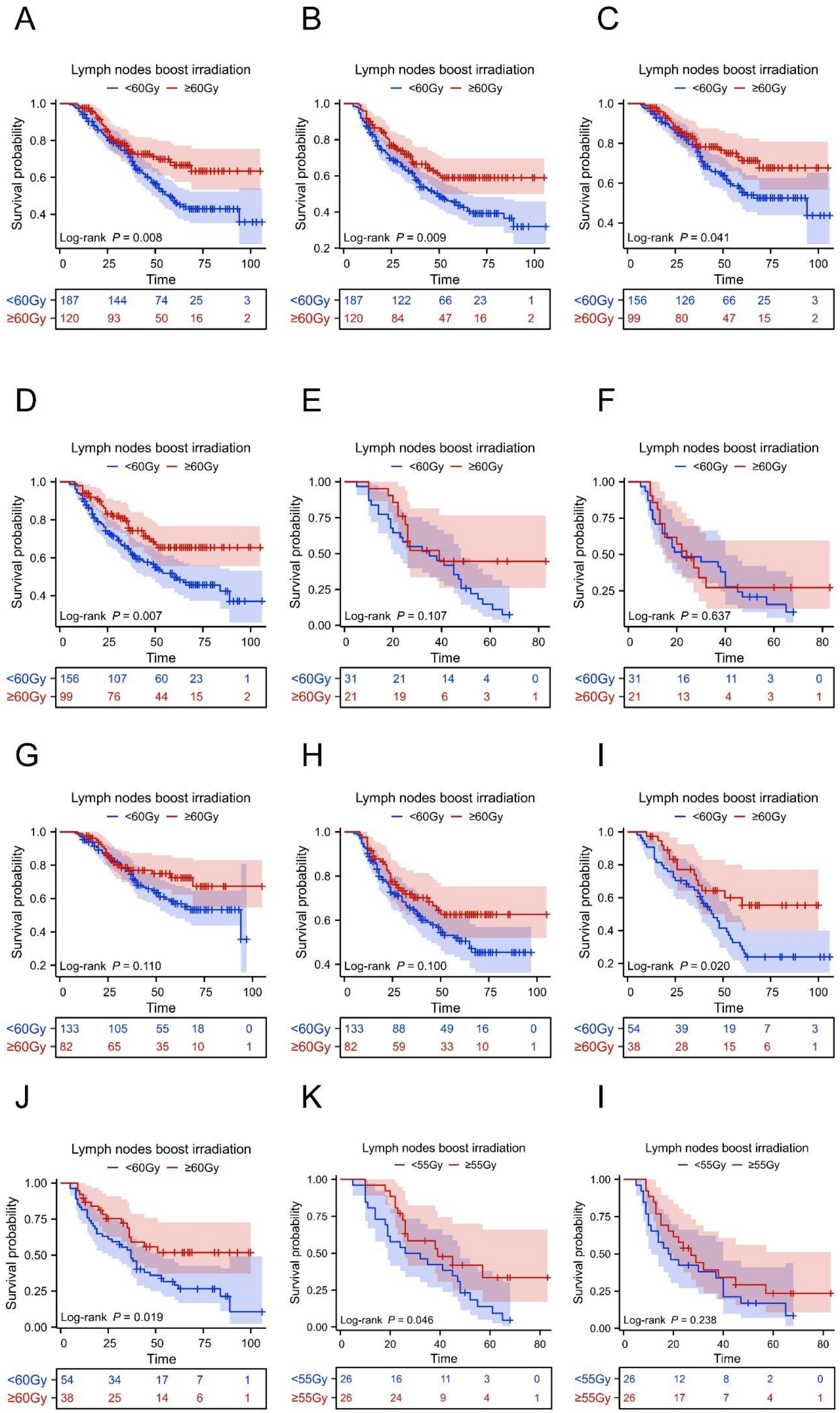

Supplementary Figure 6. (**a-b**) Kaplan-Meier analyses of LN boost irradiation ≥60 Gy EQD2 in the entire cohort for OS and PFS. Kaplan–Meier analyses of LN boost irradiation ≥60 Gy EQD2 in patients with FIGO stages IIIC1r (**c-d**) and IIIC2r (**e-f**) for OS and PFS. Kaplan-Meier analyses of LN irradiation ≥60 Gy EQD2 in patients with ≤3 positive LNs (**g-h**) and those with >3 positive LNs (**i-j**) for OS and PFS. (**k-l**) Kaplan–Meier analyses of LN boost irradiation ≥55 Gy EQD2 in patients with FIGO stage IIIC2r for OS and PFS.

OS, overall survival; PFS, progression-free survival; FIGO, International Federation of Obstetrics and Gynecology; LN, lymph node

Supplementary Figure 7. Kaplan-Meier analyses of LN boost irradiation ≥60 Gy EQD2 in patients with Node-RADS scores of 1–2 (**a-b**), 3 (**c-d**), 4 (**e-f**), and 5 (**g-h**) for OS and PFS.

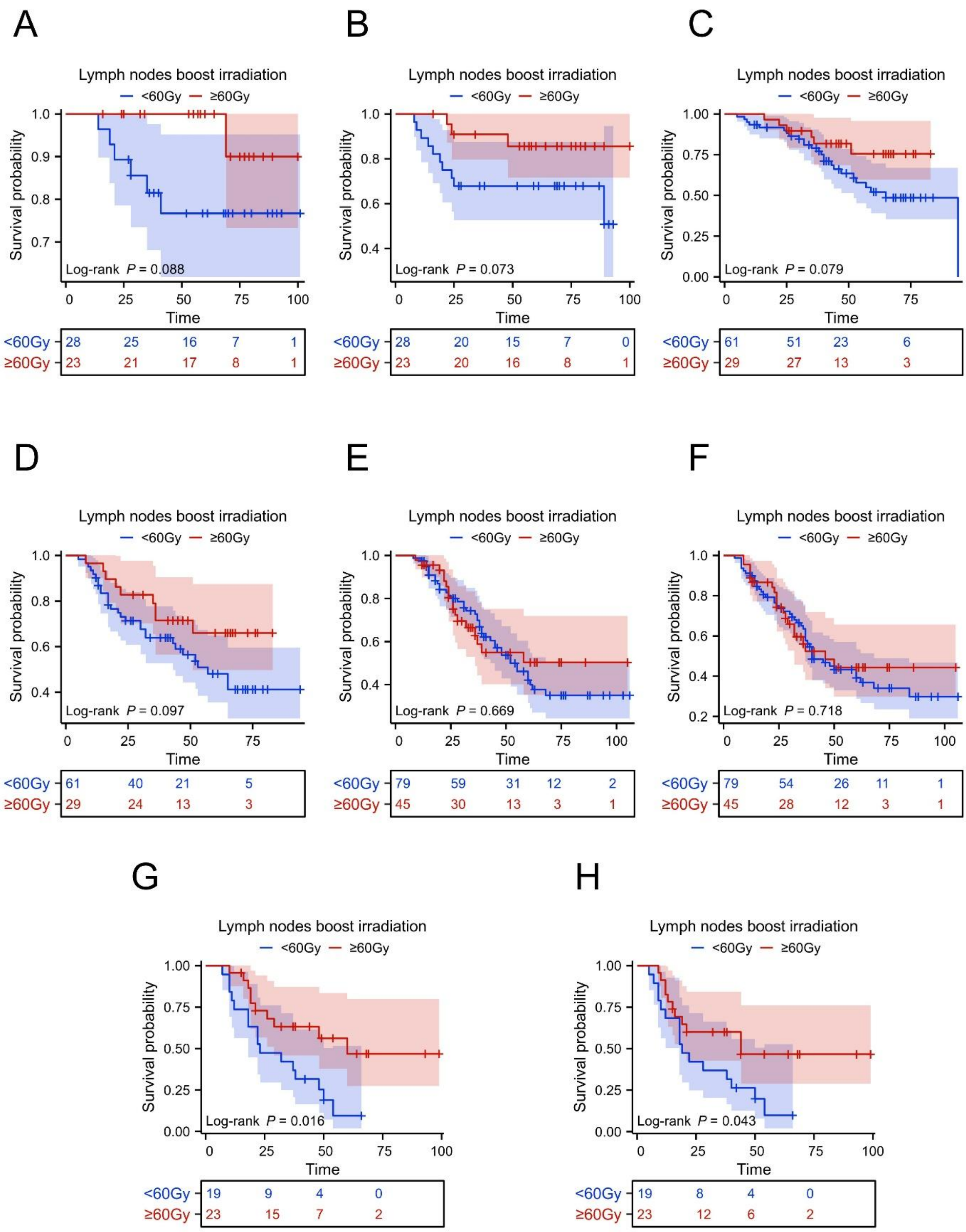

OS, overall survival; PFS, progression-free survival; Node-RADS: Node-Reporting and Data System

Supplementary Figure 8. Kaplan-Meier analyses of T stage (**a-b**), histological classification (**c-d**), primary tumor size (**e-f**), adjuvant chemotherapy (**g-h**), and tumor differentiation (**i-j**) in the entire cohort for OS and PFS.

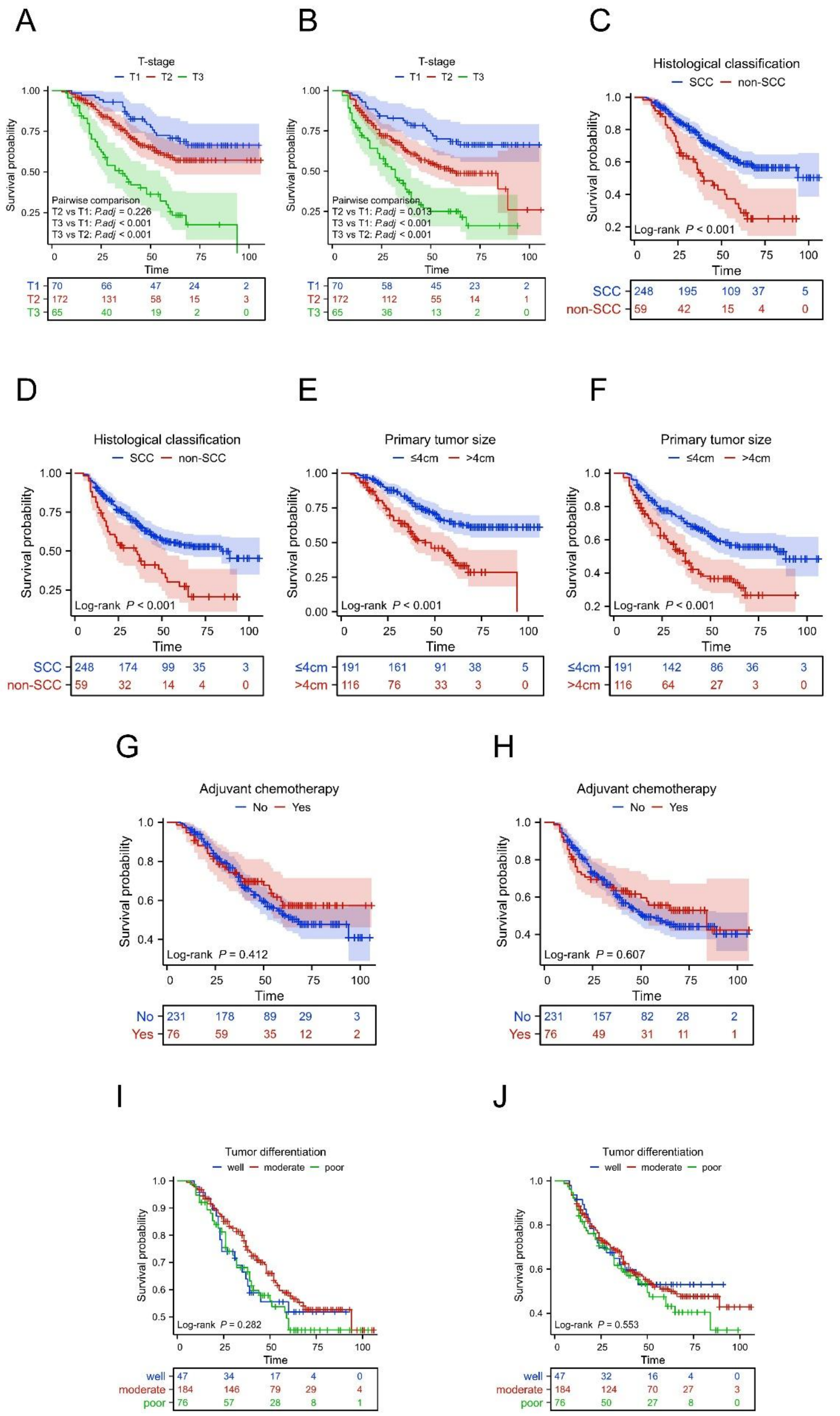

OS, overall survival; PFS, progression-free survival

Supplementary Table 1. The most relevant findings of this study

Category	Finding	Details
Study Population	Total Patients	• 307 patients with FIGO 2018 stage IIICr cervical cancer;<br>• Training Cohort: 191 patients; Validation Cohort: 116 patients<br>• 5-year OS: 54.3%; 5-year PFS: 49.4%
Independent Prognostic Factors	OS	• PALN metastasis (HR = 1.80, p = .045)<br>• Non-squamous histology (HR = 1.79, p = .032)<br>• Node-RADS score 4–5 (vs. 1–2; HR = 3.24, p = .028)<br>• LN boost dose ≥60 Gy EQD2 (HR = 0.499, p = .022)
	PFS	• T2 stage (vs. T1; HR = 2.08, p = .026)<br>• Non-squamous histology (HR = 1.96, p = .006)<br>• LN boost dose ≥60 Gy EQD2 (HR = 0.48, p = .005)
Nomogram performance	Discriminative Ability (C-index)	OS:<br>• Training cohort: 0.775; Validation cohort: 0.750;<br>PFS:<br>• Training cohort: 0.717; Validation cohort: 0.702;
	tROC analysis	the values for the 1-, 3-, and 5-year OS:<br>• Training cohort: 0.910, 0.763, and 0.837; Validation cohort: 0.787, 0.815, and 0.839<br>the values for the 1-, 3-, and 5-year PFS:<br>• Training cohort: 0.822, 0.735, and 0.795; Validation cohort: 0.828, 0.747, and 0.803
	Calibration curves	High consistency between predicted and observed outcomes for OS and PFS

	Clinical Utility (DCA)	Positive net benefit across wide threshold probabilities (outperformed FIGO staging)
	Risk Stratification	5-year OS (high-risk vs low-risk):
		• Training cohort: 28.2% vs 86.4%, *P* <.001; Validation cohort: 24.6% vs 74.5%, *P* <.001
		5-year PFS (high-risk vs low-risk):
		• Training cohort: 25.9% vs 73.7%, *P* <.001; Validation cohort: 19.5% vs 69.5%, *P* <.001
	Node-RADS Score	5-year OS: Worse with higher scores (score 1-2, 3 and 4-5; OS: 86.8% vs. 59.8% vs. 40.3%, *P* < .001)
		5-year PFS: Significant difference between scores 4–5 vs. 1–2 (37.6% vs. 75.7%, *P* < .001)
	PALN Metastasis	Worse 5-year OS (21.6% vs. 61.4%, *P* < .001) and 5-year PFS (19.4% vs. 55.7%, *P* < .001)
	>3 Positive LNs	Worse 5-year OS (38.1% vs. 62.4%, *P* < .001) and 5-year PFS (37.7% vs. 55.0%, *P* = .006)
Survival Analysis	LN Boost Dose ≥60 Gy EQD2	Improved 5-year OS (66.6% vs. 47.2%, *P* < .01) and 5-year PFS (59.0% vs. 43.6%, *P* < .01)
		· Benefit significant in IIIC1r stage, >3 positive LNs, and Node-RADS score of 5
	Node-RADS Impact	Prognostic value significant in IIIC1r stage and ≤3 positive LNs
	LN Boost Dose ≥55 Gy EQD2	improved OS (5-year: 33.49% vs. 13.85%, *P* = .046) but not PFS (*P* = 0.238) for patients with stage IIIC2 disease

Abbreviations:

FIGO: International Federation of Gynecology and Obstetrics; Node-RADS: Node-Reporting and Data System; PALN: Para-aortic Lymph Node; LN: Lymph Node; OS: Overall Survival; PFS: Progression-Free Survival; HR: Hazard Ratio; tROC: Time-dependent Receiver Operating Characteristic; AUC: Area Under the Curve; DCA: Decision Curve Analysis; EQD2: Equivalent Dose in 2 Gy fractions
